# Effective Inhibitor Removal from Wastewater Samples Increases Sensitivity of RT-dPCR and Sequencing Analyses and Enhances the Stability of Wastewater-Based Surveillance

**DOI:** 10.3390/microorganisms12122475

**Published:** 2024-12-02

**Authors:** Nico Linzner, Alexander Bartel, Vera Schumacher, José Horacio Grau, Emanuel Wyler, Henrike Preuß, Sonja Garske, Julia Bitzegeio, Elisabeth Barbara Kirst, Karsten Liere, Sebastian Hoppe, Tatiana A. Borodina, Janine Altmüller, Markus Landthaler, Martin Meixner, Daniel Sagebiel, Uta Böckelmann

**Affiliations:** 1 Laboratory of Berliner Wasserbetriebe, Berliner Wasserbetriebe, 13629 Berlin, Germanyuta.boeckelmann@bwb.de (U.B.); 2 Unit for Surveillance and Epidemiology of Infectious Diseases, State Office for Health and Social Affairs (SOHSA), 10559 Berlin, Germany; 3 Amedes Medizinische Dienstleistungen GmbH, 37081 Göttingen, Germany; 4 Max-Delbrück-Center for Molecular Medicine in the Helmholtz Association (MDC), Berlin Institute for Medical Systems Biology (BIMSB), 10115 Berlin, Germany; 5 Genomics Technology Platform, Berlin Institute of Health at Charité-Universitätsmedizin Berlin, 10178 Berlin, Germany; 6 Institut für Biologie, Humboldt-Universität zu Berlin, 10117 Berlin, Germany

**Keywords:** wastewater-based surveillance, dPCR, inhibitors of molecular biology applications, inhibitor removal, time series stability, SARS-CoV-2, sequencing, robust, noise, Germany

## Abstract

Wastewater-based surveillance (WBS) is a proven tool for monitoring population-level infection events. Wastewater contains high concentrations of inhibitors, which contaminate the total nucleic acids (TNA) extracted from these samples. We found that TNA extracts from raw influent of Berlin wastewater treatment plants contained highly variable amounts of inhibitors that impaired molecular analyses like dPCR and next-generation sequencing (NGS). By using dilutions, we were able to detect inhibitory effects. To enhance WBS sensitivity and stability, we applied a combination of PCR inhibitor removal and TNA dilution (PIR+D). This approach led to a 26-fold increase in measured SARS-CoV-2 concentrations, practically reducing the detection limit. Additionally, we observed a substantial increase in the stability of the time series. We define suitable stability as a mean absolute error (MAE) below 0.1 log_10_ copies/L and a geometric mean relative absolute error (GMRAE) below 26%. Using PIR+D, the MAE could be reduced from 0.219 to 0.097 and the GMRAE from 65.5% to 26.0%, and even further in real-world WBS. Furthermore, PIR+D improved SARS-CoV-2 genome alignment and coverage in amplicon-based NGS for low to medium concentrations. In conclusion, we strongly recommend both the monitoring and removal of inhibitors from samples for WBS.

## 1. Introduction

During the severe acute respiratory syndrome coronavirus 2 (SARS-CoV-2) pandemic [1], several countries established wastewater-based surveillance (WBS) as a powerful tool to monitor infection dynamics within the human population in a distinct catchment area. WBS advantages include a faster turnaround time, a more cost-effective approach, and less bias to testing regulations than a medical testing system [2]. During a viral infection, the nucleic acids are shed in bodily fluids such as feces, urine, and saliva, which are then collected in communal sewers [3,4,5,6]. Thus, viral RNAs, including SARS-CoV-2, Influenza viruses A and B, the Respiratory syncytial virus (RSV), and others, can be detected in wastewater. Usually, nucleic acids from 24 h composite samples of the raw influent of wastewater treatment plants (WWTPs) are extracted for WBS. Using reverse transcription-polymerase chain reaction (RT-PCR) and next generation sequencing (NGS), the SARS-CoV-2 RNA copies/L wastewater can be quantified and circulating coronavirus variants determined. Besides the sampling approach, specific PCR detection assays, and deep sequencing methods, clean and effective extractions of viral RNA are crucial for successful WBS [7]. Wastewater is a complex, cloudy, aqueous sludge, containing organic material (e.g., feces, urine, fat, detergents, phenols, etc.), inorganic compounds (metals, acids, ammonia, phosphate, nitrogen, etc.) as well as microorganisms with their proteins, polysaccharides, and nucleic acids [8]. Therefore, many substances, also called inhibitors, which are present in wastewater, can potentially inhibit enzymatic reactions in molecular biology applications, such as RT, PCR, and sequencing analysis [9,10,11], and might consequently interfere with WBS analyses.

In general, inhibitors in molecular biology applications are a heterogeneous group of chemical substances, including humic substances, fulvic acid, polysaccharides, phenols, or urea. They can interfere with primer annealing, interact with nucleic acids, inhibit or degrade the RT/PCR enzymes (reverse transcriptase, DNA polymerase), or decrease the fluorescence of the probes [10,11]. Humic acids, which originate from the decomposition of plant material and naturally occur in wastewater, are probably the most prevalent inhibitory substances [11,12,13]. Furthermore, urea, which is excreted in urine, can degrade DNA polymerases [14,15]. In feces, polysaccharides, bile salts, or urea were assumed as important inhibitors [10,16].

In the Berlin Wastewater Cluster (BEWAC), an interdisciplinary team from local public institutions, including the water company, the public health authority, and a research institute, collaborate to perform WBS. For this purpose, the three largest WWTPs in Berlin (Ruhleben, Schönerlinde, Waßmannsdorf) were sampled two to three times a week, and the international airport BER was sampled once a week since 2022. The catchment areas of the WWTPs encompass 84% of Berlin’s population (Figure S1), which is equal to approximately 3.25 million inhabitants [17].

In this study, we aim to provide an approach to identify and reduce inhibition in WBS. We show substantial inhibition effects in our reverse transcription-digital polymerase chain reaction (RT-dPCR) analyses. The inhibition results in a marked underestimation of SARS-CoV-2 RNA amounts in the communal sewers of Berlin. Additionally, we show that inhibition varies substantially with a strong dependence on the time and place of sampling. This adds variability between consecutive samples and complicates the trend analysis. Therefore, we show that the removal of inhibitors improves the stability of the time series, which enhances epidemiologic interpretation. Furthermore, inhibitor removal increases coverage and alignment in RNA sequencing. In conclusion, the inclusion of PCR inhibitor removal (PIR) during sample processing significantly improves the validity of WBS.

## 2. Materials and Methods

### 2.1. Wastewater Sampling, Total Nucleic Acid Extraction, Inhibitor Removal, and Quantification of SARS-CoV-2 RNA Copies/L in Wastewater by dPCR

Communal wastewater of three wastewater treatment plants (EU UWW Codes: Ruhleben DETP_BE01, Schönerlinde DETP_BE02, Waßmannsdorf DETP_BE03) in Berlin and the airport BER (Figure S1) was collected from raw influent by automatic samplers (WaterSam GmbH & Co. KG, Balingen, Germany). Thus, samples were taken every 2 h for a period of 24 h total. These twelve samples were subsequently mixed to a 24 h composite sample in a volume-proportional manner. Using the Wizard^®^ Enviro TNA Kit (Promega Corp., Madison, WI, USA) as a direct capture method, total nucleic acids (TNA) were extracted and concentrated from 40 mL of 24 h composite samples, resulting in a final volume of 50–100 µL following the manufacturer’s instructions [18]. Following the operating instructions, the OneStep^TM^ PCR inhibitor removal (PIR) kit (D6030, Zymo Research, Irvine, CA, USA) was used to remove inhibitors of molecular enzymatic reactions. The kit encompasses one cleaning step over a column, which efficiently retains inhibitors, including long and short chain humic acids, tannins, or polyphenols. After column preparation, 100 µL aqueous TNA solution was transferred to the column and centrifuged for 3 min at 16,000× *g*.

To quantify the RNA amounts of SARS-CoV-2 N1 and N2 target genes as well as PMMoV as a fecal surrogate indicator, dPCR analysis was performed using the QIAcuity One 5-plex dPCR system (Qiagen, Hilden, Germany), the OneStep Advanced Probe Kit (Qiagen, Hilden, Germany), and the GT digital SARS-CoV-2 Wastewater Surveillance Assay (GT Molecular, Fort Collins, CO, USA). Each RT-dPCR reaction contains 10 µL 4 × Probe Mastermix, 0.4 µL reverse transcriptase mix, 0.2 µM probe and 0.6 µM forward and reverse primers, 5 µL template TNA, and PCR-grade water in a final volume of 40 µL. Due to high PMMoV concentrations in the sewers of Berlin, the TNA extracts were diluted 100 times prior to dPCR analyses of PMMoV. The GT digital SARS-CoV-2 Wastewater Surveillance Assay (GT Molecular, Fort Collins, CO, USA) contains all primers and probes for the SARS-CoV2 N1 and N2 detection as well as PMMoV detection, and positive controls. The primers and probes for the SARS-CoV-2 targets N1 and N2 are based on the US-CDC design [19,20]. The RT-dPCR cycling program includes reverse transcription (50 °C for 30 min), RT enzyme inactivation (95 °C for 2 min), and 2-step cycling (45 cycles of denaturation at 95 °C for 10 s and combined annealing/extension at 55 °C for 30 s). In each dPCR run, positive and non-target controls were included. A 6-carboxy-X-rhodamine (ROX) hydrolysis probe for the detection of the N1 target gene and a 6-carboxyfluorescein (FAM) hydrolysis probe for the N2 target gene were used. For PMMoV detection, a FAM probe was applied. The data were analyzed using the QIAcuity Software Suite version 2.5.0.1 (Qiagen, Hilden, Germany). The SARS-CoV-2 target genes N1 and N2 as well as PMMoV RNA copies/L wastewater were calculated accordingly using the formula of the GT-digital wastewater surveillance guide (GT Molecular, Fort Collins, CO, USA).

### 2.2. Inhibition Assessment by Dilution Assays

For inhibition assessment, the QuantiNova^TM^ IC Probe assay was used following the manufacturer’s instructions (Qiagen, Hilden, Germany). Accordingly, an artificial RNA (QuantiNova^TM^ IC RNA) was spiked into PCR mixtures in the absence or presence of undiluted and diluted (1:2, 1:5, 1:10) TNA extracts to quantify the inhibition grade of wastewater samples. The QuantiNova^TM^ IC RNA is an artificial RNA of 200 bp amplicon length and can be specifically detected by the QuantiNova^TM^ IC Probe Assay (Qiagen, Hilden, Germany, Cat-No: 205813). As described above, 5 µL of undiluted TNA and 5 µL of each dilution were used in dPCR. To further analyze the potential occurrence of inhibitors, the extracted TNA without or after PIR treatment (D6030, Zymo Research, Irvine, CA, USA) were diluted with nuclease-free water, as follows: 1:2, 1:5, 1:10, and 1:20.

### 2.3. Sequencing of SARS-CoV-2 RNA of Wastewater Samples and Data Analysis

For the sequencing of SARS-CoV-2 RNA in wastewater, we generated amplicon-based short-read sequencing libraries. We used the COVIDseq kit (Illumina, cat# 20049393) with the v4 primer pool (Illumina, cat# 20065135) spiked with the 11 supplementary primers, as described in the Illumina technical note “Guidelines for detecting the SARS-CoV-2 Omicron variant using the Illumina COVIDSeq Test”. Libraries were prepared and pooled in batches of 20–37 samples, according to the manufacturer’s instructions. To increase multiplexing possibilities, some samples were indexed with the DNA/RNA UDI Set B (Illumina cat# 20091656) and not with the Set A index plate that comes with the COVIDseq kit. Sequencing was conducted on a NextSeq 2000 device using a P1 300 cycles kit (Illumina, cat# 20050264). Reads were mapped to SARS-CoV-2 via a bowtie2-samtools pipeline.

### 2.4. Statistical Analysis: Flow Normalization and Stability Estimation

All statistical analyses were performed in R version 4.4.1 (R Foundation Vienna). Confidence intervals were estimated using bootstrap with a 95% confidence level. SARS-CoV-2 RNA copies/L were log10-transformed to achieve normal distribution. Subsequently, the values were normalized dividing by a flow correction factor, which is calculated as the sampling flow rate divided by the dry weather flow rate (Table S1).

As a quality parameter, we use the stability of the time series over consecutive samples (also known as detrended between-sample variance). First, we estimated the non-linear trend of the normalized measurements using a penalized thin plate spline with one degree of freedom (k) per 28 days (length of time series) using the R package mgcv version 1.9-1 [21]. We calculated the detrended values (also known as residuals or errors) by subtracting the estimated trend from the observed data. To quantify the stability, we calculated the mean absolute error (MAE) of the residuals. We chose MAE because it measures the average magnitude of the errors without giving extra weight to occasional larger deviations, and therefore focuses more on overall stability rather than sporadic anomalies [22]. Since the MAE was calculated using base-10 logarithmic values, we can back-transform it into the geometric mean relative absolute error (GMRAE) by calculating 10 MAE, which represents the error on a percentage scale [22]. A lower MAE and GMRAE indicate greater stability of the samples over time, reflecting reduced noise and higher data quality. Recommendations for acceptable MAE thresholds, further methodological assumptions, and the benefits of this approach are provided in the Discussion section (Section 4.2).

## 3. Results

### 3.1. Dilutions of the TNA Extracts Reveal a High Inhibitory Effect of the TNAs Derived from Berlin’s WWTPs

Raw wastewater in Berlin is a dirty and murky sludge–water mix (Figure S2, Table S1), containing many potential inhibitors. To ensure a valid WBS analysis, it is important to extract nucleic acids in a careful and clean manner. We used the direct capture method for the concentration and extraction of the TNA (Promega Corp., Madison, USA), which results in the highest dPCR-measured concentration of SARS-CoV-2 compared to ultrafiltration, affinity-based beads, and solid extraction [23]. However, we particularly observed a slightly yellowish stain in our TNA extraction in autumn and winter when increased rainfall occurs. Additionally, we detected comparatively low SARS-CoV-2 RNA loads for a large city, like Berlin (3.8 Mio inhabitants), with each of the three WWTPs serving several hundred thousand up to more than a million inhabitants in their catchment areas.

**Figure 1:**
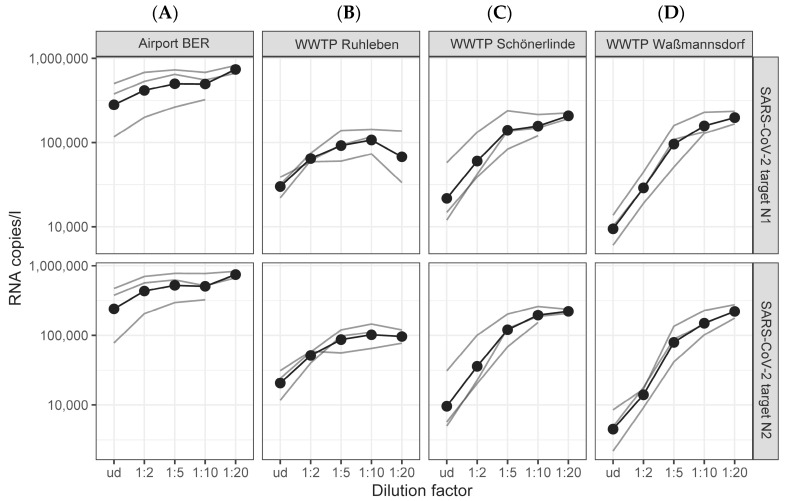
Dilutions of TNA extracted from the airport BER (**A**) and the WWTPs Ruhleben (**B**), Schönerlinde (**C**), and Waßmannsdorf (**D**) display an increase in the SARS-CoV-2 target N1 and N2 copies after multiplication with the corresponding dilution factors, indicating the presence of PCR inhibitors. Data show three biological replicates of each WWTP and the airport from July and August 2023. TNA extracts were measured undiluted (ud) and diluted up to 1:20 (one replicate only up to 1:10). Black dots and lines show the geometric mean of the three biological replicates, individual replicates are indicated as a gray line.

To further examine this apparent discrepancy, we investigated the potential presence of inhibitors in our TNA samples. It is recommended to use either an inhibition control (IC) or serial dilutions of the nucleic acid extracts to monitor inhibition [24]. However, our dPCR system and the commercial wastewater primer–probe kits did not include any IC. Therefore, we prepared serial dilutions up to 1:20 of the TNA extracts and measured the dilutions in comparison to the undiluted extract as a template in dPCR runs. We expected the RNA amount after dilution factor recalculation to be nearly equivalent in all samples. However, the SARS-CoV-2 RNA values of the target N1 and N2 genes were enhanced in all dilutions of wastewater samples compared to the corresponding undiluted TNA extracts (Figure 1). Remarkably, it was necessary to dilute the samples of the WWTPs up to 1:20 (Figure 1B–D) to detect no further increase in the calculated RNA copy numbers. Furthermore, the measured RNA copies of the SARS-CoV-2 target N1 were roughly twice as high as for the target N2 in the undiluted samples, whereas increasing dilution factors resulted in equalizing the N1 and N2 RNA copies. These results clearly show that the TNA extracts of the WWTPs contain inhibitors. The sample of the airport BER did not require such high dilutions (up to 1:5) (Figure 1A), indicating that the wastewater of the BER is less affected by inhibitors.

### 3.2. The Combined Usage of Inhibitor Removal and Dilution (PIR+D) Lead to Enhanced Amplification and an Equal N1/N2 Target Ratio

**Figure 2:**
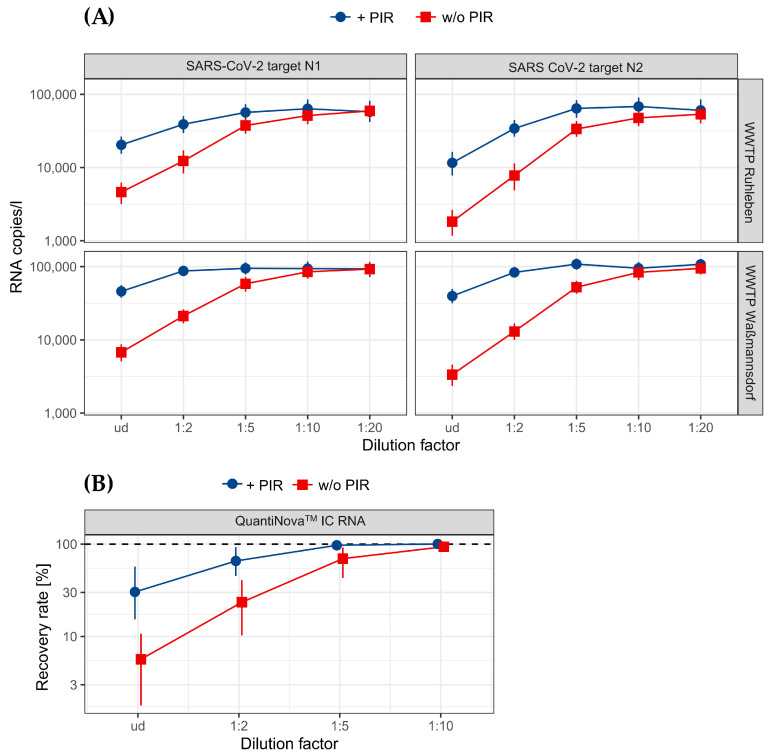
PCR inhibitor removal (PIR) leads to improved dPCR runs and increased detection of target RNA copy numbers. (**A**) The data show comparative dPCR analyses using TNA extracts of the WWTPs Ruhleben (*n* = 21) and Waßmannsdorf (*n* = 19) from 4 February 2024 to 27 March 2024 with (blue, circle) or without (red, square) PIR treatment as templates. PIR was conducted using dilutions and the OneStep^TM^ PCR inhibitor removal kit. (**B**) QuantiNova^TM^ IC RNA was spiked into dPCR mixtures in the presence of PIR-treated (blue, circle) or untreated (red, square) TNA extracts from WWTP Ruhleben samples (*n* = 4). The quantified QuantiNova^TM^ IC RNA copy number in the absence of WWTP TNA extracts was set to 100% and the data show the calculated relative amounts of amplified QuantiNova^TM^ IC RNA in the presence of WWTP TNA extracts.

**Figure A1:**
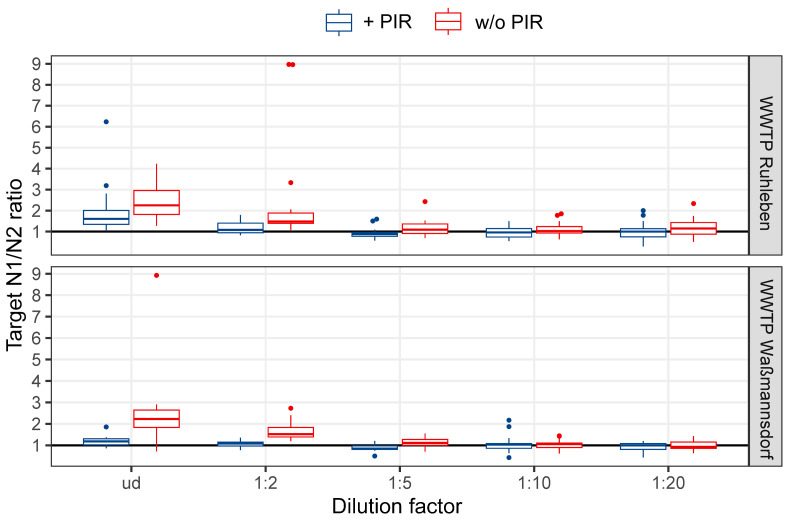
The N1/N2 ratio converges at decreased PCR inhibitor concentrations. The ratios were calculated after comparative dPCR analyses using TNA extracts of the WWTPs Ruhleben (*n* = 21) and Waßmannsdorf (*n* = 19) from 4 February 2024, until 27 March 2024, with (in blue, left) or without (in red, right) PIR treatment as templates. PIR was conducted using the OneStep^TM^ PCR inhibitor removal kit.

While inhibition can be reduced using dilutions, lower dilutions in the range of 1:2 to 1:5 should be preferred over higher dilutions, like 1:10 or 1:20, to avoid an excessive increase in the detection threshold [7]. To decrease the required dilution levels of our WWTP TNA extracts, we added an inhibitor removal step (PIR), utilizing the OneStep^TM^ PCR inhibitor removal kit (D6030, Zymo Research, Irvine, CA, USA). Subsequently, we prepared dilutions of TNA extracts treated or untreated by PIR and compared the quantified RNA values of the SARS-CoV-2 target N1 and N2 genes. Here, we detected a substantial increase in RNA copy numbers after PIR even in the undiluted TNA extracts (Figure 2A, Figure A1, Figure S3 and S4). For example, we detected only 9750 RNA copies/L for N1 and 3435 copies/L for N2 in the WWTP Waßmannsdorf without PIR, whereas these numbers increased after PIR to 60,750 copies/L for N1 and 57,890 copies/L for N2, respectively (Figure S4). The values of the N1 and N2 target genes were almost equal after PIR (ratio N1/N2 around 1) (Figure A1, Figure S3B,D and S4B,D) compared to the TNA extracts without PIR (ratio N1/N2: >2) (Figure A1, Figure S3A,C and S4A,C). The lower measured N2 copy numbers compared to N1 gene values indicate that the N2 target gene or its primers are more susceptible to inhibitors. Interestingly, the amounts of detected N1 and N2 target RNA copies were nearly equal using the 1:5 dilution of the TNA extracts without PIR (Figure A1). In addition, the combined usage of the PIR kit and dilution (PIR+D) further increased the RNA copy numbers (Figure 2A), suggesting that the combination of PIR cleaning and dilution (PIR+D) is necessary to sufficiently reduce inhibitors from TNA extracts. Moreover, by applying PIR to the sample preparation process, we reduced the required dilution factor from up to 20 (without PIR) to 5 (with PIR) (Figure 2A, Figure A1, Figure S3 and S4).

To confirm the presence of inhibitors in our TNA extracts, we spiked artificial QuantiNova^TM^ IC RNA (Qiagen, Hilden, Germany) into PCR mixtures in the presence of TNA extracts derived from the WWTP Ruhleben with or without PIR treatment. As a control, the QuantiNova^TM^ IC RNA was also quantified in nuclease-free water and the copy number was set to 100%. As expected, the amplified IC RNA copy number was strongly reduced in the presence of the undiluted WWTP TNA extracts (Figure 2B). Recovery was approximately 38% in the presence of PIR-treated TNA, but only 8% of the IC RNA could be detected with untreated TNA extracts. The dilution of TNA extracts at 1:5 improved recovery for PIR-treated samples to 97% and to 75% for untreated samples (Figure 2B). These results clearly confirm the presence of a substantial amount of inhibitors in our extracted TNA and reveal that a 1:5 dilution combined with the PCR inhibitor removal kit (PIR+D) is sufficient to reduce the inhibition to a minimum.

Since huge concentrations of PMMoV have been measured in the TNA extracts of our sampling sites, we had to dilute the TNA extracts 1:100 prior to the PMMoV dPCR analyses. This high dilution level already led to a strong reduction in inhibitor concentration (Figure S5). Since the PMMoV RNA copy numbers did not change due to the PIR treatment, inhibitor removal is not required at these dilution levels.

### 3.3. PIR Led to an Increased Stability of WBS

**Figure 3:**
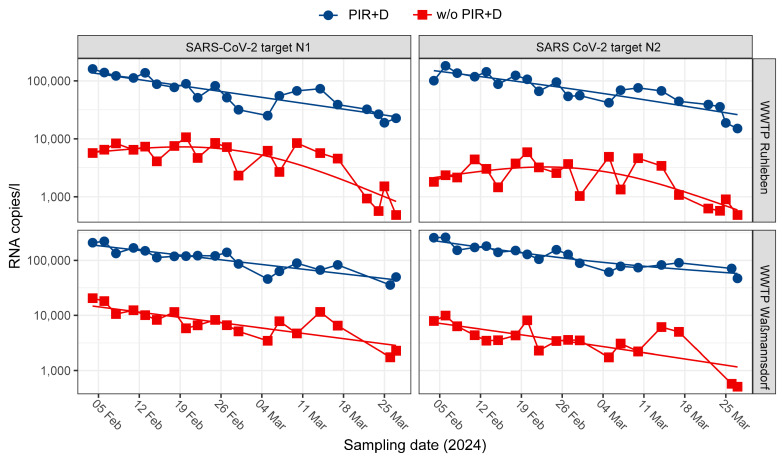
Comparison of the flow-normalized SARS-CoV-2 RNA values (mean of the targets N1 and N2) measured by dPCR between TNA extracts with PCR inhibitor removal and five-fold dilution (PIR+D) (blue, circle) or undiluted TNA without PIR (w/o PIR+D) (red, square) derived from Ruhleben (*n* = 21) and Waßmannsdorf (*n* = 19). The data show a comparative analysis of all samples (2–3 per week) from 4 February 2024, until 27 March 2024.

Next, we investigated the effects of PIR over a longer period of time in a realistic wastewater surveillance setting using all samples (2–3 per week) from the two largest WWTPs in Berlin from February to March 2024. Based on the results above, we defined sufficient inhibitor removal as the combined cleaning of the TNA extract using the OneStep^TM^ PCR inhibitor removal kit (Zymo Research, Irvine, CA, USA) and a subsequent five-fold dilution (PIR+D). The measured SARS-CoV-2 N1 and N2 copy numbers with or without PIR+D were normalized using the flow of the WWTP. Afterwards, we compared the results of dPCR analyses of the untreated, undiluted TNA extracts with PIR+D samples (Figure 3). For both WWTPs Ruhleben and Waßmannsdorf, the SARS-CoV-2 N1 and N2 target loads were approximately 26 times higher after PIR+D compared to the RNA target copy numbers measured without cleaning and dilution (Figure 3). The consecutive dPCR measurements showed a higher stability after PIR+D than those using TNA extracts without PIR. While the mean absolute error (MAE) was 0.219 (GMRAE 65.5%) without PIR, the MAE was substantially reduced to 0.097 after PIR treatment (GMRAE 26.0%). These results indicate that PIR substantially improves the time series stability of the measurements.

**Figure 4:**
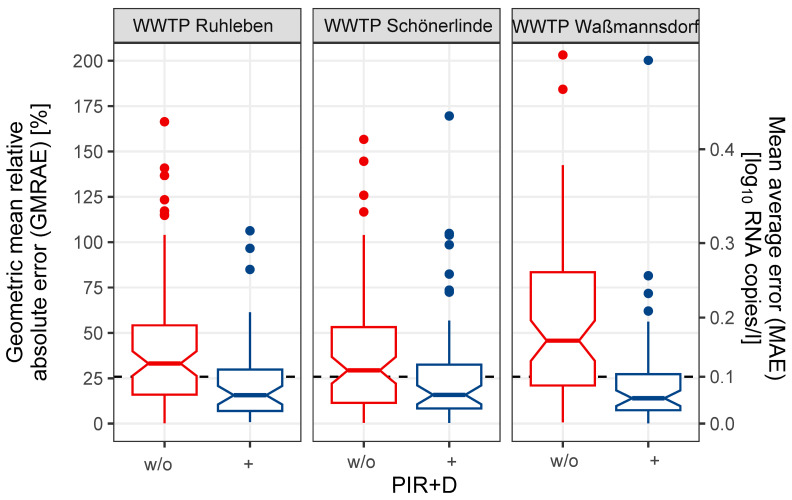
PIR+D treatment increases stability of wastewater monitoring. To exclude seasonal effects, we assessed time series stability over comparable periods from April to October. Samples treated with the new inhibitor removal protocol (PIR+D) are from 2024 and samples without PIR+D treatment (w/o) are from 2023. The dashed line indicates our stability threshold of an MAE of 0.1 (GMRAE 26%); after implementing PIR+D, all wastewater treatment plants (WWTPs) fell below this threshold, demonstrating improved stability. The number of observations differs due to a lower sampling frequency in 2024: WWTP Ruhleben (PIR+D: *n* = 50, w/o: *n* = 80), Schönerlinde (PIR+D: *n* = 54, w/o: *n* = 80), Waßmannsdorf (PIR+D: *n* = 53, w/o: *n* = 78). For full times series of measurements and fit of the spline, see Figure A2.

**Figure A2:**
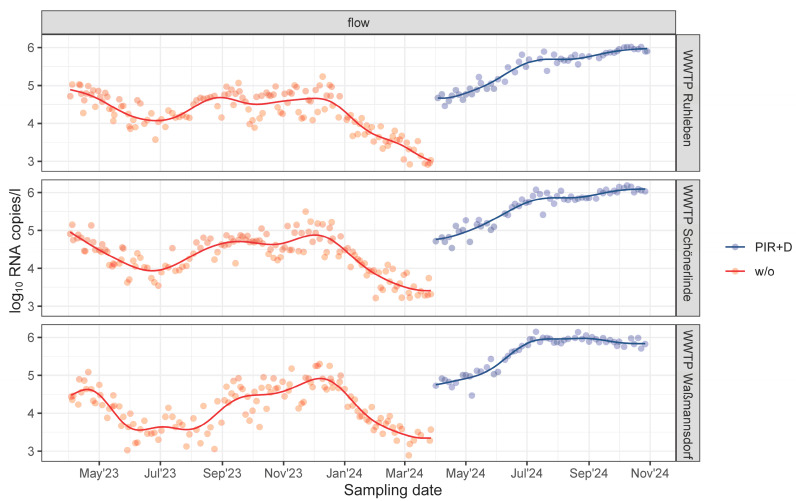
Full times series of measurements (points) from April 2023 to October 2024, with fitted splines (lines) for visualization of trends. Measurements performed without PIR (w/o, red), from 1 April 2023 to 31 March 2024, show lower concentrations and greater variabillity, while those conducted with PIR+D (blue), from 1 April 2024 onwards, show higher concentrations and improved stability, as indicated by reduced deviations from the trend lines.

After implementing the new inhibitor removal protocol (PIR+D) into routine wastewater surveillance in April 2024, our goal was to determine whether the improved stability observed during the testing phase in February and March 2024 would persist over longer periods in a real-world setting (full time series of data, see Figure A2). Based on the previous data, we chose a mean absolute error (MAE) of less than 0.1 as our stability quality threshold, which corresponds to a geometric mean relative absolute error (GMRAE) of less than 26%. With our new protocol (PIR+D), the MAE of all WWTPs was successfully reduced below 0.1 (Figure 4, Table S2), with the largest reduction observed in Waßmannsdorf from a MAE of 0.186 (GMRAE 53.6%) to a MAE of 0.081 (GMRAE 20.4%), mirroring the results of the initial tests (Figure 2). Therefore, the stability of the time series of SARS-CoV-2 RNA measurements was improved long term by PIR+D.

### 3.4. PIR Improves Coverage and Alignment of NGS

**Figure 5:**
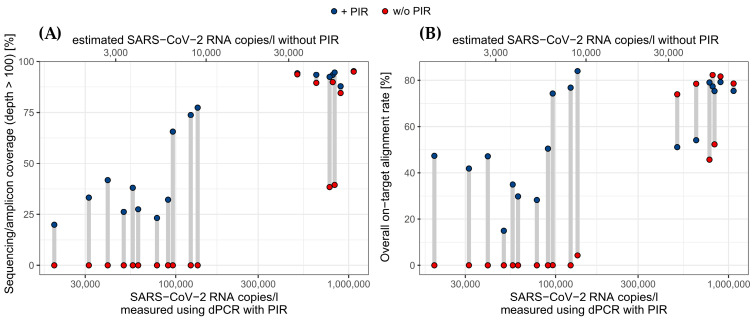
SARS-CoV-2 sequencing quality (coverage and alignment) with and without PCR inhibitor removal (PIR). Sequencing with PIR (blue) and w/o PIR (red) was performed on the same wastewater sample (connected by a gray line). (**A**) Coverage of the SARS-CoV-2 genome in % of genome with sequencing/amplicon depth above 100. (**B**) Overall, on-target alignment rate as the % of reads that mapped the reference genome.

After seeing substantial improvements in the PCR-based detection of SARS-CoV-2 RNA upon removal of inhibitors, we investigated whether this procedure also enhances high-throughput sequencing of viral RNA from wastewater. Indeed, when SARS-CoV-2 RNA levels were low, the sequencing coverage and depth could be improved by PIR (Figure 5A,B and Figure S7). For high SARS-CoV-2 levels, sequencing quality was mostly high with 100% coverage, but still 2 of 7 high RNA concentration samples showed problems during sequencing when inhibitor removal was not performed (w/o PIR). All eleven low SARS-CoV-2 level samples showed an improvement in coverage after being PIR treated, but did not reach 100% genome coverage. Regarding the on-target alignment rate, all low SARS-CoV-2 concentration samples improved after treatment. Among the seven high-concentration samples, three showed similar results with and without PIR treatment. The two samples that had lower coverage without PIR also exhibited lower alignment rates when untreated. Notably, two high-concentration samples showed a decrease in the alignment rate after PIR treatment, but this did not seem to affect their sequencing coverage, which remained close to 100%.

## 4. Discussion

### 4.1. Influence of PIR on dPCR and Next Generation Sequencing (NGS) Analyses

In this study, we have ascertained that our total nucleic acids (TNA) extracts derived from raw influent of WWTPs in Berlin are strongly polluted with inhibitory substances, which severely impair molecular analyses, including dPCR and NGS. We developed an effective inhibitor removal (PIR) procedure by combining a PCR inhibitor removal kit (Zymo Research) with subsequent five-fold dilution (PIR+D). PIR alone substantially improved the dPCR and sequencing analyses (Figure 2A, Figure 5A,B and Figure S7), revealing the kit’s suitability to reduce inhibition. The manufacturer’s instructions refer to an efficient removal of polyphenolic compounds, humic and fulvic acids, tannins, melanin, and others by using the kit (Zymo protocol, version 2.0.2, 2024). However, only applying the kit to TNA extracts is insufficient for removing inhibitors. With our modification (PIR+D), we were able to further reduce inhibition levels, demonstrating the necessity for this additional step and the combined approach. This was confirmed using artificial IC RNA (Figure 2B), which shows that this inhibition is not SARS-CoV-2-specific. Hence, we conclude that the PIR kit is limited to specific inhibitor subclasses and removal performance is potentially hindered by high inhibitor concentrations, as suggested by recent studies [25,26]. Since NGS is also based on a similar workflow (RT and qPCR, Artic PRimer Kit), it is also affected (Figure 5 and Figure S7) and improved by PIR.

While some loss of TNA by using PIR is expected, this is negligible compared to the increase in sensitivity we observed. The PMMoV measurements were unaffected, i.e., showed no TNA loss (Figure S5). It has also been previously shown that the TNA loss is negligible [27]. The kit we used is currently the most affordable, easiest, and fastest [27], but this still results in a small cost increase per sample. Apart from commercial PIR kits, adsorbents, like bovine serum albumin (BSA) or polymers (e.g., DAX-8), can be used for inhibitor removal. However, BSA can degrade after several PCR cycles or is sterically inhibited by the molecules present in wastewater [26,28]. Further research is needed to determine, which PIR method performs best, but the efficiency of each PIR method is dependent on the composition of inhibitors [10], which is expected to vary widely between WWTPs.

Inhibitors usually present in wastewater samples pose a significant problem for molecular analyses, such as dPCR and NGS. While this issue can be partly mitigated by performing a serial dilution of up to 1:20 or above [29,30,31,32], these dilution levels often result in an increased limit of detection. This, in turn, may lead to underestimating the target, thus giving a false representation of the actual viral load within the wastewater under scrutiny. Consequently, it is advantageous to use only minimal dilution factors within samples prior to analysis [7,10,25,26]. Moreover, taking the above mentioned limitations of PIR kits on certain inhibitor subclasses into account, we showed that only a combined approach is adequate to sufficiently remove inhibitors. A five-fold dilution was suitable for all of our samples, while Waßmannsdorf’s samples could even be used with only a two-fold dilution (Figure 2A, Figure 3, Figures S3 and S4).

Furthermore, the inhibition of the N2 target was significantly higher, resulting in a N1/N2 ratio of approximately 2 in untreated samples. After applying PIR+D, however, we showed that the ratio changed to 1, indicating that inhibition was reduced to a minimum (Figure 2A and Figure A1). This suggests that the N1/N2 ratio can be an indicator of enzyme inhibition for SARS-CoV-2 in wastewater-derived samples.

### 4.2. Time Series Stability in Wastewater Surveillance

We recommend using the time series stability as a quality parameter for infectious disease wastewater monitoring. We assume that any difference in normalized viral concentrations between consecutive samples should mainly reflect the trend, such as gradual increases or decreases in SARS-CoV-2 levels. Fluctuations beyond this trend are likely due to measurement variability rather than actual changes in viral shedding in the population.

PIR+D improved the stability as measured by the MAE below our threshold of 0.1 log_10_ RNA copies/L and the GMRAE below 26% (Figure 4). This directly improves the epidemiological interpretation because less noisy measurements allow for easier and faster trend detection for improved interpretation of infection dynamics [33]. Alternatively, if fast detection is not crucial, the improved stability may allow for less frequent sampling since fewer data points are needed to estimate trends. In our case, improving the stability allowed us to reduce the sampling frequency from three to two samples per week, thus reducing cost.

Alongside methodological improvements, stability analysis can show whether normalization effectively compensates for short-term fluctuations, such as those caused by rainfall. If the stability of measurements after normalization is not higher than for non-normalized measurements, the normalization does not sufficiently reduce short-term variability. Normalization could still adjust for long-term seasonal biases, but research on this topic is limited. Seasonal changes found for PMMoV levels are mostly interpreted as an undesirable effect [34].

We recommend assessing stability primarily from samples with a viral load sufficiently above the limit of quantification to reduce irrelevant noise potentially introduced by time periods between disease outbreaks. Additionally, WWTPs which serve smaller populations may exhibit greater stochastic variability [2]. For trend estimation in wastewater monitoring, in addition to splines, other methods like LOESS (locally estimated scatter plot smoothing regression) can also be used [35,36]. Independent of the method for the non-linear trend estimation, overfitting should be avoided, since it leads to an underestimation of the MAE [37]. COVID-19 waves typically last multiple months. Therefore, the trend estimation should reflect this and not show short-term trend reversals (Figure A2).

Our cutoff of an MAE below 0.1 log_10_ RNA copies/L (GMRAE < 26%) could also be applicable to other pathogens measured by wastewater monitoring, which we plan to investigate further.

### 4.3. Possible Inhibitory Modes of Action in the dPCR Analyses

Having established that inhibition processes play an essential part in dPCR analyses of wastewater samples, the question remains as to which mechanisms and substances are predominantly causing the observed effects. Due to the complex structure of wastewater, it can be assumed that a high diversity of inhibitors with different inhibitory modes of action exist, including fluorescence quenching, impairment of primer annealing, interaction with nucleic acids, and enzymatic inhibition of reverse transcriptase or DNA polymerase [10,11].

One of the most common PCR inhibition mechanisms is the degradation or direct inhibition of DNA polymerase [10,11,25,38]. Many substances potentially present in wastewater, such as urea, humic, fulvic and tannic acids, melanin, or bile salts, can cause inhibitory effects, leading to delayed amplification signals in qPCR analyses [39]. In dPCR, such inhibition results in intermediate partitions between positive and negative signals. However, our 1D scatter plots for both PIR(+D)-treated and untreated samples showed a clear separation, with no intermediate partitions detected (Figure S6). This indicates that the DNA polymerase was not degraded or delayed by PCR inhibitors.

Another mechanism of inhibitors is the fluorescence quenching of the probes caused by substances, including humic acid or an altered ion content [11,38]. However, humic acid quenches the fluorescence of DNA-binding dyes like SYBR Green, but not hydrolysis probes due to the oligonucleotide binding [40,41]. Using ROX and FAM hydrolysis probes, we observed no reduced fluorescence signals without PIR (Figure S6), indicating that fluorescence quenching is unlikely.

However, we did detect a higher reduction in the N2 target amplification than for the N1 target (Figure 1, Figure 2A and Figure A1), indicating that the amplification of target gene N2 was more susceptible to inhibitors compared to N1. This suggests that the present inhibitors do not affect the targets or the primer equally. Hence, we assume that nucleic acid interaction or hindering of primer annealing inhibit the PCR. A similar issue was previously observed by da Silva and colleagues [42].

The reverse transcriptase as the first enzyme of the RT-dPCR reaction is susceptible to inhibitors [9,43,44]. While we did not observe any inhibitory effects during DNA polymerase activity (no intermediate partitions as shown in Figure S6), and even the artificial IC RNA was not fully amplified using untreated extracts, we postulate that the main inhibition occurs during the reverse transcription process. Mitranescu et al. recently overcame inhibition by the addition of multiple different reverse transcriptases, further underlining our findings of RT inhibition playing a more prominent role than polymerase inhibition [45].

### 4.4. Impacts on Inhibitor Concentrations, Workflow Considerations for Wastewater Monitoring, and Possible Inhibitory Modes of Action in Berlin

Due to the dynamic and complex structure of wastewater, the inhibitor concentration and composition are dependent on many factors. These include the size of the catchment area, the present sewer system (separate or combined), the discharge of industrial wastewater, and the environment [46,47]. Thus, we assume that the influences of the specific substances occurring at each sampling site are substantial. For example, SARS-CoV-2 copy numbers were already substantially higher in the samples of the airport BER before inhibitor removal, and dilution had less of an effect as compared to WWTP samples, indicating less inhibition.

This observation might be due to the sampling site being a direct pipeline from Berlin’s international airport without additional environmental and industrial input, or feces. Additionally, the catchment area of the airport is substantially smaller than for the WWTPs, as is shown in Figure S1. While the raw influent of the WWTP Ruhleben exclusively contains urban wastewater from approximately 1.7 million inhabitants, the catchment areas of the WWTPs Schönerlinde and Waßmannsdorf also include rural wastewater derived from Brandenburg. (Figure S1, Table S1). Furthermore, wastewater treated in Ruhleben is derived mainly from a combined sewer system, whereas the other two WWTPs receive wastewater primarily from separated systems. Consequently, inhibitor concentrations and compositions vary at each site and fluctuate daily.

Inhibitor concentrations and compositions may fluctuate additionally due to seasonal changes and weather effects, such as increased precipitation in winter contributing to higher inhibitor influx from roads and traffic. Moreover, many respiratory viruses increasingly appear in the fall and winter season, including influenza viruses and SARS-CoV-2 [48,49]. The increased number of infection cases could lead to elevated levels of pharmaceuticals, like antiviral substances, entering the sewer [50,51,52]. These substances may well affect RT-dPCR and NGS reactions.

The whole wastewater monitoring workflow should be robust against inhibitors. By using a direct capture method, higher yields can be achieved [23]. Moreover, we quantify the viral load using dPCR, which has been shown to be less susceptible to PCR inhibitors [24,53,54] than qPCR. Nevertheless, as we have shown with our study, PCR inhibition remains an issue even with dPCR. Therefore, it is strongly recommended to assess inhibition by using an internal inhibition control (IC) or by serial dilutions [7,10,11].

Many inhibitory substances have been already characterized in environmental and in clinical fecal samples, including humic, fulvic and tannic acids, urea, hemoglobin, polysaccharides, bile salts, immunoglobulin G (IgG), polyphenol, metal ions, detergents, melanin, or antiviral substances [10,11]. It is highly likely that many of those named substances are also present in wastewater. Many RT inhibitors, including zidovudine, nevirapine, stavudine, and lamivudine, are frequently used to treat human immunodeficiency virus (HIV) infections and can be detected in raw wastewater [50,51]. In particular, humic acid is proposed as one of the most prevalent PCR inhibitory substances because it naturally exists in environmental water matrices and has been known to impair RT activity [12,13]. Humic acid and, in high concentrations, fulvic acid are able to inhibit the RT similar to the often-used RT inhibitory drug zidovudine (azidothymidine) [55,56]. The yellowish stain of TNA extracts as an indicator of the occurrence of humic substances has been already shown previously [57]. Since our TNA extracts without PIR treatment occasionally had a slightly yellowish stain, the co-extraction of humic acid from wastewater is likely, strengthening the hypothesis that the RT step is one of the main targets of inhibition.

Furthermore, it has been previously shown that organic humic substances, including humic acids, as the main PCR inhibitory substances in environmental samples, can also directly bind to RNA and DNA molecules [39,58,59], suggesting a direct interaction between humic substances and the nucleic acids of the extracts. Moreover, IgG is well known as a PCR inhibitor in clinical samples, which has as its proposed mode of action the binding to single-stranded DNA (ssDNA), possibly interfering with primer annealing. This effect also plays a role in dPCR [41,60]. Hence, humic acid or IgG could cause the difference between the N1 and N2 targets’ amplification.

We illustrated in this study that solely using the PIR kit was not sufficient to remove the inhibitory effects, resulting in the incomplete amplification of IC RNA. This is most likely due to the high concentration and composition of inhibitors in Berlin’s wastewater, most notably humic substances and pharmaceuticals. This could explain the necessity of the further five-fold dilution of the TNA extracts to sufficiently reduce inhibition in our WBS workflow.

## 5. Conclusions

Wastewater is a complex mixture of substances originating from (I) the environment, (II) the industry, and (III) the human [8]. Hence, many substances are included in wastewater, which can affect and complicate molecular analyses, especially RT-dPCR and consequently NGS sequencing [25,26]. As a result, the spike-in of an inhibition control (IC) is recommended as a best practice for wastewater monitoring by the EU Joint Research Centre and the US National Academies of Sciences [61,62]. In this study, we confirm that our TNA extracts derived from raw influent of WWTPs in Berlin are strongly polluted with inhibitory substances, which severely impair the dPCR and sequencing analyses. The importance of inhibition control and removal in wastewater monitoring is exemplified by the following two challenges:

1. Inhibitory substances increase the effective detection limit.
2. The concentrations and composition of inhibitory substances vary between wastewater samples due to several factors.

Challenge (1) would pose a smaller issue for PCR quantification, if the concentration and composition of inhibitory substances were constant across all samples. In this case, the bias introduced by inhibition would be constant, affecting primarily the detection limit. But this increased detection limit also impacts the SARS-CoV-2 sequencing substantially. For early warning, the detection of new circulating SARS-CoV-2 sublineages through sequencing during low incidence periods is essential [17,63]. 

However, due to challenge (2), the inhibition introduced can differ widely, even for samples collected on consecutive days. To evaluate the measurement errors introduced by short-term changes in inhibitory substance concentration and composition, we introduce time series stability as a quality parameter in infectious disease wastewater monitoring.

To effectively address both challenges, we recommend the use of commercial PIR kits combined with dilution (PIR+D) as an effective inhibitor removal strategy for analysis of wastewater samples.

## Data Availability

All data supporting the findings of this study are available within the paper and its Supplementary Information. Raw sequencing data has been deposited at the NCBI GEO data repository under the accession number GSE283001.

## Supplementary Materials

The following supporting information can be downloaded at: https://www.mdpi.com/article/10.3390/microorganisms12122475/s1, Figure S1: Wastewater-based surveillance in Berlin; Figure S2: Raw influent wastewater; Figure S3: Comparison of TNA extracts of WWTP Ruhleben, Figure S4: Comparison of TNA extracts of WWTP Waßmannsdorf, Figure S5: PMMoV comparison, Figure S6: Exemplified 1D scatter plots of dPCR, Figure S7: Coverage sequencing. Table S1: Characterization of raw influent wastewater of the WWTPs Ruhleben, Schönerlinde and Waßmannsdorf. Data of TKN, TP, BSB5 and CSB are calculated averages at dry weather from 2024. Table S2: Stability of WBS for each treatment plant with and without PIR+D for April to October. Data from 2023 was used for w/o and data from 2024 for PIR+D.
