# Supplementary material for: Effective Inhibitor Removal from Wastewater Samples Increases Sensitivity of RT-dPCR and Sequencing Analyses and Enhances the Stability of Wastewater-Based Surveillance
Source: Microorganisms. 2024 Dec 2;12(12):2475. doi: 10.3390/microorganisms12122475 (PMC11728302; doi:10.3390/microorganisms12122475)
Supplement: Supplementary file 1 [file microorganisms-12-02475-s001.zip › microorganisms-3328409-supplementary.pdf]

Supplemental material of research article:

Effective inhibitor removal from wastewater samples increases sensitivity of dPCR and sequencing analyses and enhances the stability of wastewater-based surveillance

Nico Linzner [1,†], Alexander Bartel [2,*,†], Vera Schumacher [1], José Horacio Grau [3], Emanuel Wyler [4], Henrike Preuß [1], Sonja Garske [2], Julia Bitzegeio [2], Elisabeth Barbara Kirst [4,5], Karsten Liere [3], Sebastian Hoppe [2], Tatiana A. Borodina [4,5], Janine Altmüller [4,5], Markus Landthaler [4,6], Martin Meixner [3], Daniel Sagebiel [2] and Uta Böckelmann [1]

1 Laboratory of Berliner Wasserbetriebe, Berliner Wasserbetriebe, 13629 Berlin, Germany;
2 Unit for Surveillance and Epidemiology of Infectious Diseases, State Office for Health and Social Affairs (SOHSA), 10559 Berlin, Germany;
3 Amedes Medizinische Dienstleistungen GmbH, 37081 Göttingen, Germany;
4 Max-Delbrück-Center for Molecular Medicine in the Helmholtz Association (MDC), Berlin Institute for Medical Systems Biology (BIMSB), 10115 Berlin, Germany;
5 Genomics Technology Platform, Berlin Institute of Health at Charité-Universitätsmedizin Berlin, 10178 Berlin, Germany
6 Institut für Biologie, Humboldt-Universität zu Berlin, 10117 Berlin, Germany
* Correspondence: alexander.bartel@lageso.berlin.de
† These authors contributed equally to this work.

Supplemental Table S1. Characterization of raw influent wastewater of the WWTPs Ruhleben, Schönerlinde and Waßmannsdorf. Data of TKN, TP, BSB_5 and CSB are calculated averages at dry weather from 2024.

	Averages at dry weather				
	**Daily flow [m³/d]**	**Total Kjeldahl nitrogen (TKN = organic N + NH_4-N) [kg/d]**	**Total phosphorus (TP) [kg/d]**	**Biochemical oxygen demand in 5 days (BSB_5) [kg/d]**	**Chemical oxygen demand (CSB) [kg/d]**
**WWTP Ruhleben**	247,500	16,934	2,550	68,022	179,827
**WWTP Schönerlinde**	105,000	9,108	1,082	38,886	99,567
**WWTP Waßmannsdorf**	230,000	17,179	1,894	80,420	191,777

Supplemental Table S2. Stability of WBS for each treatment plant with and without PIR+D for April to October. Data from 2023 was used for w/o and data from 2024 for PIR+D.

	**n**		**MAE**		**GMRAE**	
	**w/o**	**PIR+D**	**w/o**	**PIR+D**	**w/o**	**PIR+D**
**WWTP Ruhleben**	80	50	0.140	0.085	38.0%	21.7%
**WWTP Schönerlinde**	80	54	0.126	0.093	33.8%	23.8%
**WWTP Waßmannsdorf**	78	53	0.186	0.081	53.5%	20.4%

Supplemental Figures

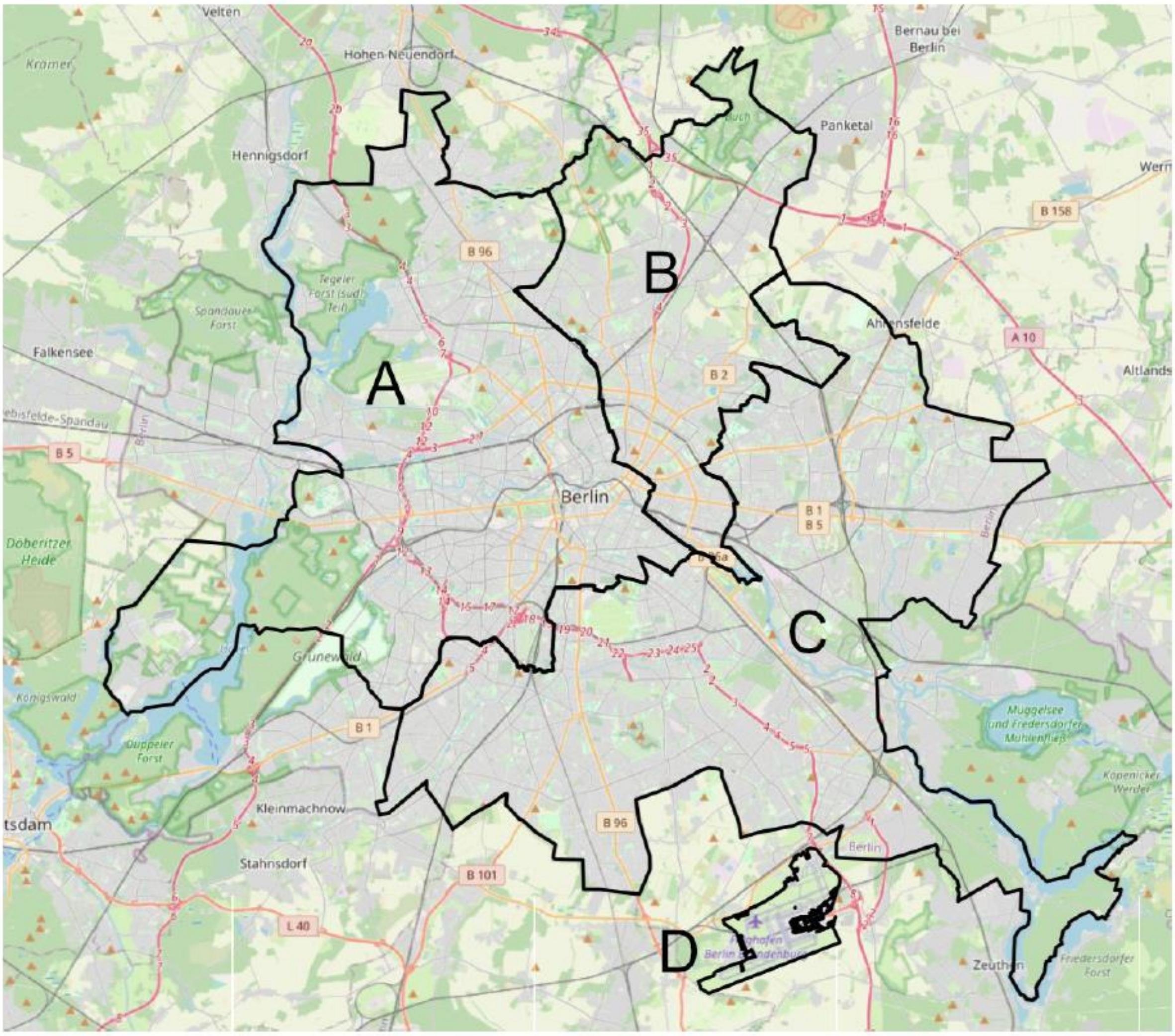

Figure S1. Wastewater-based surveillance in Berlin. Samples are taken from the three wastewater treatment plants (WWTP) **(A)** Ruhleben, **(B)** Schönerlinde as well as **(C)** Waßmannsdorf and from **(D)** the airport BER. The map shows the catchment areas of each WWTP. In total 84% of Berlin's population are captured by sampling the three WWTPs for the wastewater monitoring.

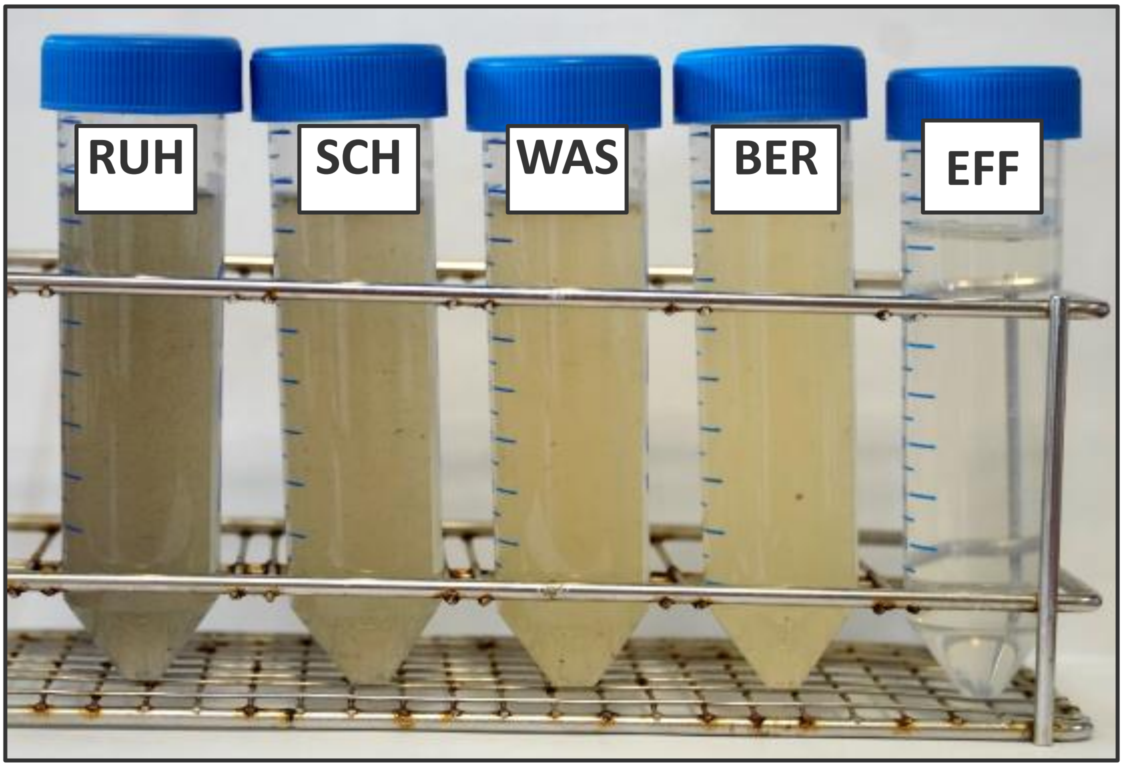

Figure 2. Raw influent wastewater of the wastewater treatment plants (WWTP) Ruhleben (RUH), Schönerlinde (SCH) and Waßmannsdorf (WAS) as well as a sample of the airport Berlin-Brandenburg (BER). For comparison, a water sample of cleaned WWTP effluent (EFF) is shown.

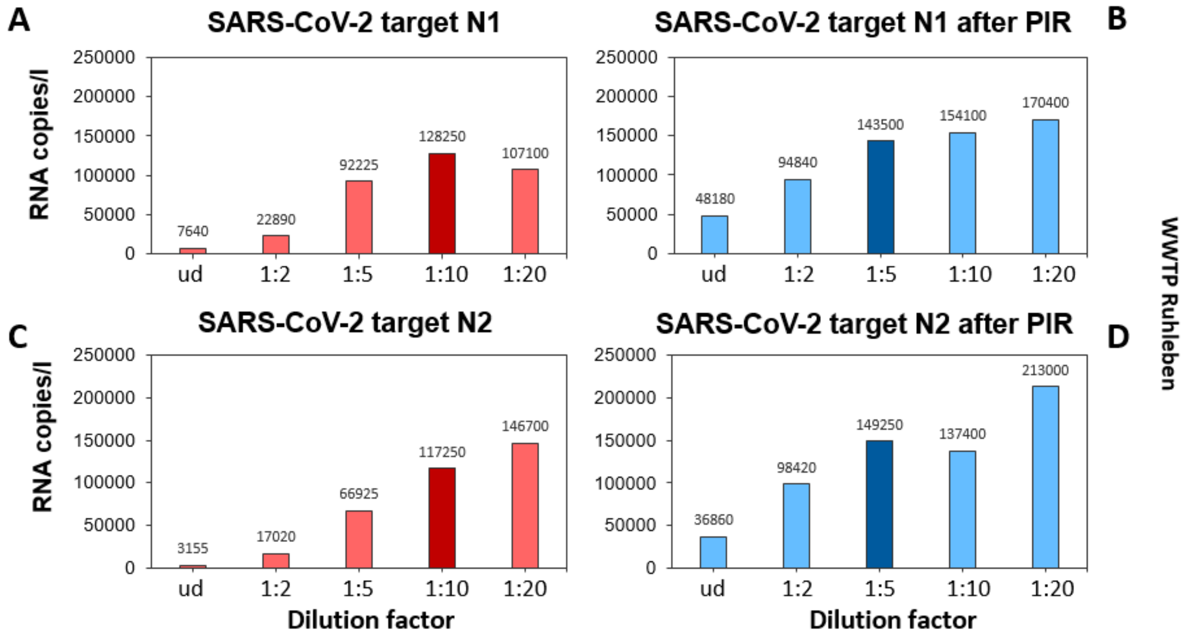

Figure S3. Comparison of TNA extracts with PCR inhibitor removal (PIR) treatment **(B, D)** show an increase of SARS-CoV-2 target RNA's of the N1 **(A, B)** and N2 **(C, D)** gene copies after recalculation of the dilution factor. The data show representative dPCR analysis of the WWTP Ruhleben from February 13th 2024. TNA extracts were cleaned with the PIR kit and were diluted up to 1:20. The dPCR analyses were conducted in comparison to TNA extracts without PIR.

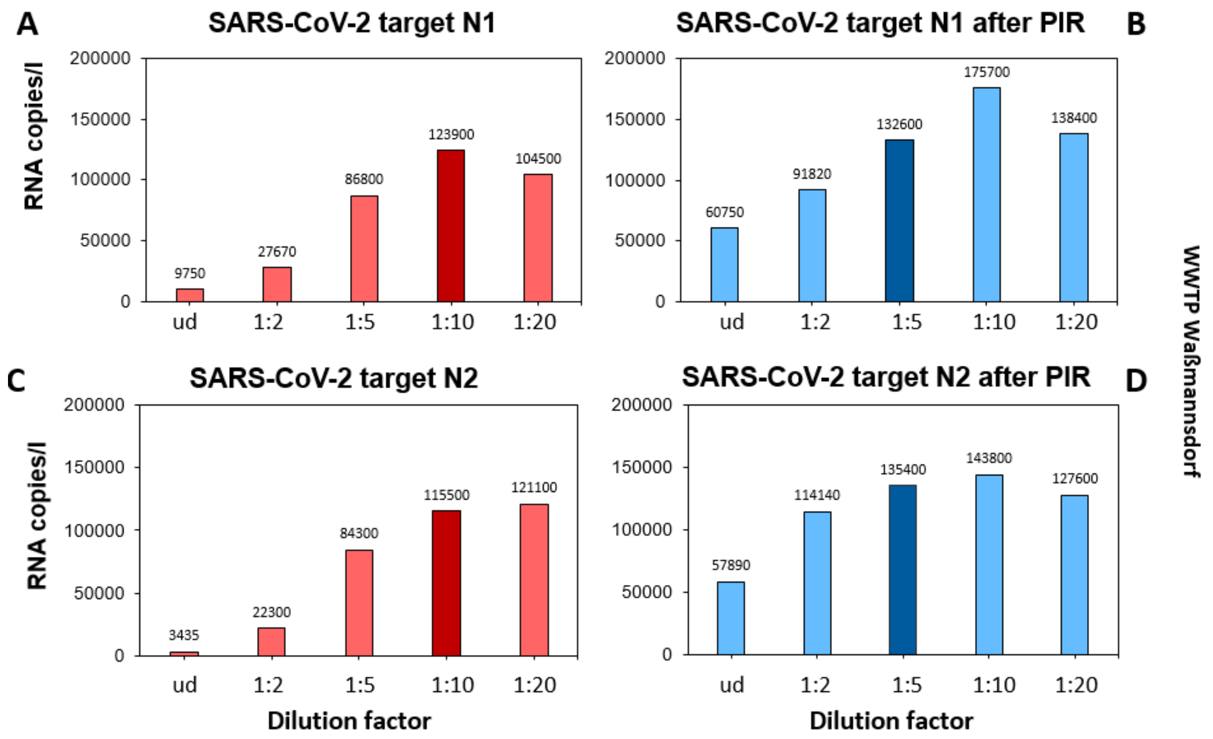

Figure S4. Comparison of TNA extracts with PCR inhibitor removal (PIR) treatment **(B, D)** show an increase of SARS-CoV-2 target RNAs of the N1 **(A, B)** and N2 **(C, D)** gene copies after recalculation of the dilution factor. The data show representative dPCR analysis of the WWTP Waßmannsdorf from February 11th 2024. TNA extracts were cleaned with the PIR kit and diluted up to 1:20. The dPCR analyses were conducted in comparison to TNA extracts without PIR clean-up.

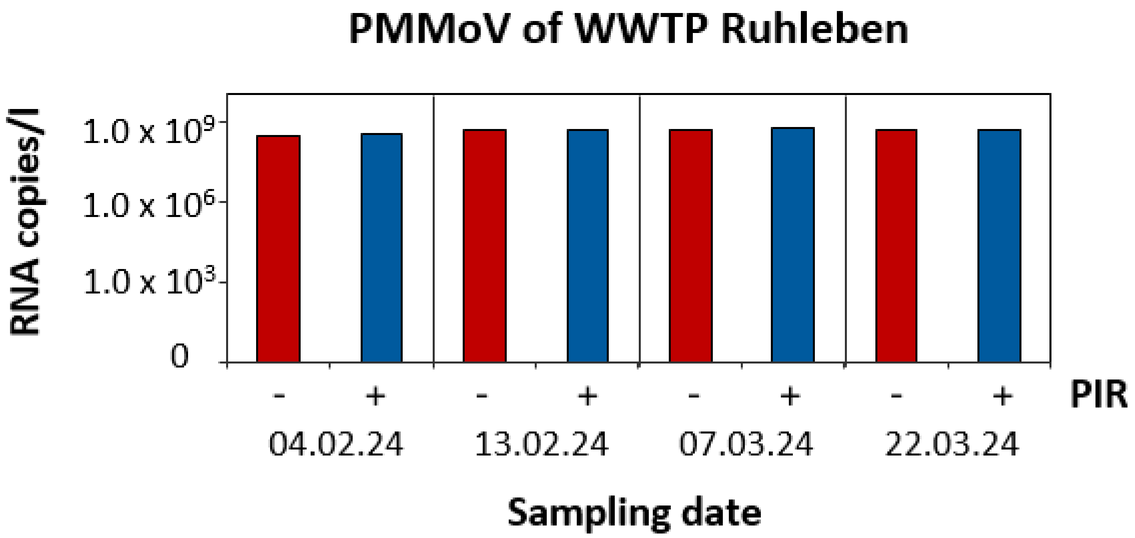

Figure S5: Comparison of dPCR analyses of PMMoV RNA from the WWTP Ruhleben indicates no inhibitory effect of the RT-dPCR. The TNA extracts were diluted 1:100 prior dPCR runs. TNA extracts were cleaned with the PIR kit (in blue). The dPCR analyses were conducted in comparison to TNA extracts without PIR clean-up (in red). The high dilution level also diluted inhibitors, therefore there is no effect of the PIR treatment. Additionally, there is no TNA loss due to the PIR treatment.

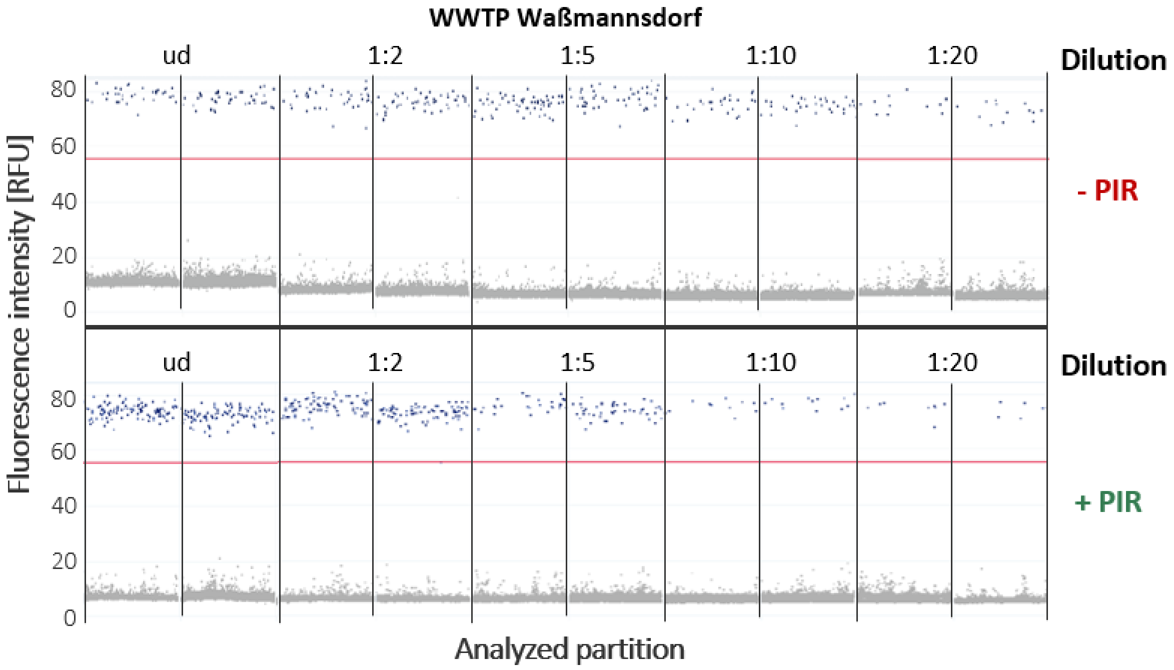

Figure S6. Exemplified 1D scatter plots of dPCR runs reveal clear differentiation between positive (in blue) and negative (in grey) partitions without intermediate partitions and increased positive partition numbers after PCR inhibitor removal (PIR) using undiluted TNA extracts as a template. Data show an example from the WWTP Waßmannsdorf.

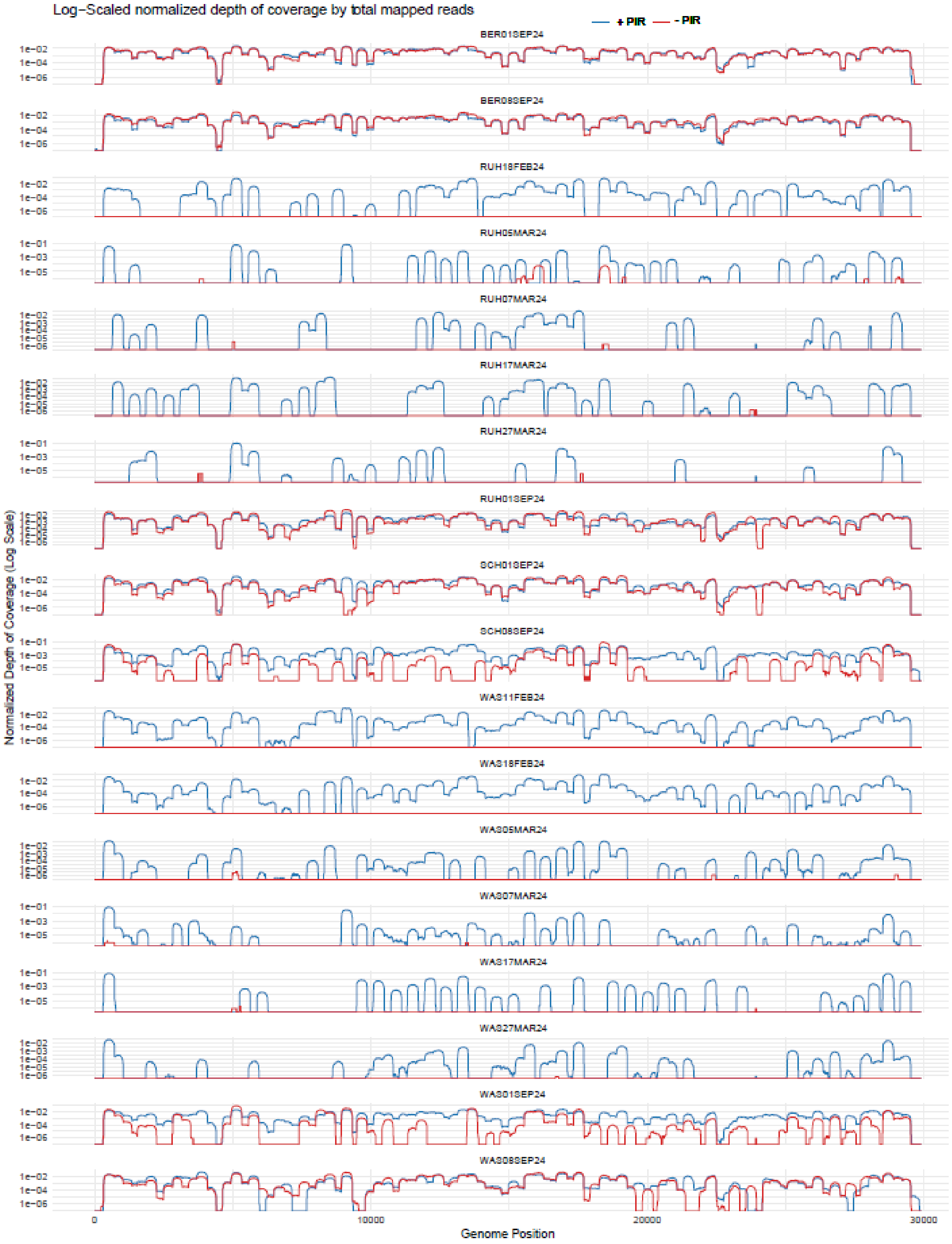

Figure S7. Coverage sequencing. TNA extracts were cleaned with the PIR kit (in blue). The NGS analyses were conducted in comparison to TNA extracts without PIR clean-up (in red).
